# Support, needs and expectations of family caregivers regarding general practitioners – results from an online survey

**DOI:** 10.1186/s12875-021-01381-4

**Published:** 2021-03-03

**Authors:** Julian Wangler, Michael Jansky

**Affiliations:** Centre for General and Geriatric Medicine, University Medical Centre Mainz, Am Pulverturm 13, 55131 Mainz, Germany

**Keywords:** Family caregivers, General practitioners, Identification, Stresses, Needs, Care, Support

## Abstract

**Background:**

Family caregivers are often the first line of support for people requiring care; although they may personally stand to benefit, these activities substantially increase the risk of physical and emotional stress. General practitioners (GPs) may provide important support and stabilisation, but need to adjust to the needs and expectations of this group in order to do so. The aim of the study was to compare the needs of family caregivers from GPs to the support they actually experience. Additional aims included determining the main factors affecting satisfaction amongst family caregivers with support from GPs. The results were used to develop possible approaches towards optimisation within the purview of general medical practice.

**Methods:**

Between January and July 2020, 612 people supporting or caring for a family member responded to an online survey posted in seventeen internet forums focused on family caregivers. In addition to the descriptive analysis, a *t*-test with independent samples was used to identify significant differences between two groups. We also used binary logistic regression analysis to identify indications of potentially influential factors regarding the experienced support from GPs.

**Results:**

Around three out of every four respondents (72%) consulted GPs in care matters. The respondents gave positive responses on their GP’s knowledge of the care situation (71%), approachability in various issues connecting with care and service towards the caregiver (82%). GPs’ efforts in meeting the needs and requirements of the care recipient were also rated positively (82%). Weaknesses in support from GPs mainly involved the lack of information on advice and assistance services (55%) as well as frequently not identifying or involving caregivers as such soon enough (42%). Results from regression analysis show that the last two aspects play a major role in subjective satisfaction amongst family caregivers with support from GPs.

**Conclusions:**

We recommend that GPs undergo further training to reinforce awareness that the care triad of needs, requirements and stresses amongst family caregivers also plays a vital role in care outcomes. With this in mind, general practice staff should adopt a pre-emptive strategy towards approaching family members about potential issues and informing them about existing assistance and support services.

**Supplementary Information:**

The online version contains supplementary material available at 10.1186/s12875-021-01381-4.

## Background

The European Union has more than 130 million people aged 60 or older; the proportion of very old people is rapidly growing [1]. This involves a constantly growing need for care and support. Some 3.4 million people in Germany were classified as requiring care at the end of 2017; around 5 million receive informal care and support according to estimates [2–4].

Care is mostly administered by individual caregivers in a domestic setting; most of them provide this long-term care to persons in need without charge [2, 5]. More than 16% of 40 to 85-year-olds provide regular assistance to at least one person in coping with everyday life according to the German Ageing Survey [6, 7]. The responsibility is often shared amongst family caregivers, female family members usually bearing the brunt of it [8, 9].

Some studies have shown that the act of caregiving may involve a subjective sense of purpose, responsibility and lowered risk of mortality [10–13]. Even so, shouldering this responsibility often entails a high level of subjective stress with increased health risks. Family care correlates far higher to mental health issues such as high levels of distress and depression than it does to physical conditions [14–18]. Some caregivers are overwhelmed with the disease-specific decisions they have to make if they have not made the appropriate preparations and preventive measures have not been taken [8, 19–24].

GPs play an important role in supporting family caregivers [25, 26]. Due to their position in the German health care system, they perform extensive primary care tasks. GPs are the first point of contact for patients and therefore often familiar with their patients and the patients’ family members for many years; there is a trusting doctor-patient relationship [5, 25, 27]. A survey of doctors in the German National Association of Statutory Health Insurance Physicians (Kassenärztliche Bundesvereinigung) concluded that 59% of family caregivers consult GPs on their care responsibilities [28].

Apart from diagnosis and treatment of health problems, GPs are in a position to provide information, advice and (emotional) support by talking to family caregivers as well as to gauge the situation and anticipate potential future needs for care [2, 27–29]. In order to support GPs in dealing with family caregivers, the German College of General Practitioners and Family Physicians (DEGAM) developed the guideline “Family Caregivers” [30].

Recognising the needs of family caregivers in time allows steps to be taken to ensure stabilisation and quality of life conducive to effective care. Providing information on support and assistance services (e.g. care centres, outpatient mental health services, dementia networks) plays a major role in this [30–33]. GPs are able to help family members prepare for the consequences of diseases such as dementia by arranging for advisory services or encouraging family members to use psychosocial support services. This also helps prevent burnout amongst caregivers and crises in a care situation [24].

The main challenges for the primary care setting in dealing with family caregivers relate in particular to the early identification of caring relatives, the splitting of caregivers and those in need of care on different physicians, the knowledge of the individual care situation as well as information and advice activities. However, only a few studies have been presented on these aspects to date. Various authors explain why identifying family caregivers early may pose a challenge to GPs: Caring relatives often do not see themselves as caregivers, but rather define themselves primarily in relation to the person being cared for (e.g. spouse, child) [9, 26, 33–35]. In addition, family carers do not always talk to their own family doctor about their care situation, which occurs especially when the family member being cared for does not have the same GP as the family caregiver [8]. Furthermore, GPs tend to place priority on the care recipient so that they may lose sight of the health and psychosocial situation of the family caregiver [27, 35–37]. In addition, the research literature addresses the problem that GPs are not always sufficiently familiar with the individual care situation of caregivers, which among other things is related to time and resource problems [24, 26]. While various support services aimed at mitigating the negative effects of home care have been established, caregivers use these services rarely [23, 24, 28].

Overall, there are currently no studies that allow a broader picture of the extent to which the problems mentioned occur in everyday practice in GP care. This study therefore takes up these challenges and examines them from the perspective of caregivers. However, it goes beyond that by exploring the needs, expectations and experiences of family caregivers with regard to GP’s support.

### Research interest

Since there is a lack of reliable studies on the type and degree of support from GPs or desires and expectations from family members being cared for, the aim of this exploratory survey was to elucidate the need for support, specifically focusing on GP care. The research interest is concentrated in following issues:

- The importance of support from GPs for family caregivers
- The support requirements of family caregivers with respect to GPs
- The extent to which support from GPs matches the requirements, desires and expectations of family caregivers respectively the challenges and problems family caregivers are confronted with
- Indications of influential factors regarding the experienced support from GPs that might have a moderating effect on the satisfaction of family caregivers with the GP’s support
- Any potential to optimise support given by GPs

## Methods

### Study design

The study was designed as an Internet-based survey of (informal) family caregivers. Researchers have shown that online surveys as a method for collecting data can achieve a lot of informative value, representativeness and quality, especially with regard to specific target groups. For instance, Gosling et al. [38] compared a large Internet sample with a set of 510 published traditional samples, showing a high degree of diversity of the Internet sample with regard to sociodemographic variables such as gender, socioeconomic status, geographic region, and age. In addition, the authors state that Internet surveys are not adversely affected by nonserious or repeat responders compared to traditional methods.

The investigation is part of a broader context, *DemStepCare,*^1^ a model project for outpatient medical and nursing care in dementia, itself part of a the broader innovation fund project supported by the German Federal Joint Committee [39].

### Survey method

The questionnaire was based on desk research, results from a focus group with eight GPs, and meetings with family caregivers (see Multimedia Appendix 1). Developing the questionnaire involved generating a catalogue of items relevant to practical support for family caregivers from GPs. Thirty-two clear-cut items were created towards developing the two key item sets (questions 14 and 15) and then reduced to the most important statements according to the opinion and experience of the doctors involved.

The two item sets were then placed one after the other in the questionnaire for the respondents. They were then asked to state which aspects of support from GPs were most important to them. Further on, these items were transferred to the personal experience to elucidate the extent to which the respondents’ expectations and desires were reflected in the reality of the support they received (see Table 1). The problems and challenges that were found in the course of the literature search with regard to the GP’s support of family caregivers (e.g. identification of caregivers in everyday practice, addressing specific needs of caregivers and individual care settings) were integrated into the item sets.

**Table 1:** Care support needs from general practitioners compared to actual support by importance (answer categories: *Very/moderately important* or *completely agree/somewhat agree*)

*It’s important to me that the general practitioner...*	Very important/ Moderately important (*N* = 612)	*The general practitioner I consult in care matters...*	Completely agree/ Somewhat agree (N = 438)
... is familiar with everyday life and challenges of caring for family members.	85%	... is familiar with everyday life and challenges of caring for family members.	72%
… is familiar with my personal situation as a caregiver.	80%	… is familiar with my personal situation as a caregiver.	71%
… feels responsibility for the issues facing people caring for family members and provides advice and assistance.	85%	… feels responsibility for the issues facing people caring for family members and provides advice and assistance.	82%
… does not wait for me to raise issues with the GP but proactively addresses issues.	68%**	… does not wait for me to raise issues but proactively addresses them.	18%**
… provides emotional support such as encouragement during care.	57%*	… provides emotional support such as by giving encouragement during care.	23%*
… makes decisions with me on caring for the person in my care.	74%*	… makes decisions with me on caring for the person requiring care.	48%*
… sees not only the needs of the person in my care but also my opinions, needs and stresses.	76%	… sees not only the needs of the person in my care but also my opinions, needs and stresses.	60%
… includes the person in my care in decisions and responds to his or her requests.	83%	… considers the person in my care in decisions and responds to his or her requests.	82%
… encourages me to raise my own health concerns.	32%	… encourages me to address my own health concerns.	23%
… has enough time for me.	85%**	… has enough time for me.	29%**
… performs home visits to relieve me and get to know the person in my care in our home environment.	75%**	… performs home visits to relieve me and get to know the person in my care in our home environment.	23%**
… gives me time to consider important decisions on care.	59%	… gives me time to consider important decisions on care.	81%
… tells me about local support and assistance services.	85%*	… tells me about local support and assistance services.	55%*
… makes arrangements for support and assistance services for me.	83%**	… makes arrangements for support and assistance services for me.	6%**
… advises me on legal aspects such as power of attorney, care assistance, and driving.	80%**	… advises me on legal aspects such as power of attorney, care assistance, and driving.	13%**
… conducts regular follow-up observations.	70%*	… conducts regular follow-up observations.	41%*
… makes diagnoses early.	78%*	… makes diagnoses early.	51%*
… manages and provides medical treatment to my family member.	52%	… manages and provides medical treatment to my family member.	45%
… arranges doctors specialised in the field for us.	85%	… arranges doctors specialised in the field for us.	95%
… explains the situation to the person in my care in ways that he or she can understand, therefore supporting me.	85%	… explains the situation to the person in my care in ways that he or she can understand, therefore supporting me.	85%
… is early to acknowledge me as the caregiver with the awareness that I am responsible for caring for my family member.	85%**	… is early to acknowledge me as the caregiver with the awareness that I am responsible for my family member’s care from the beginning.	42%**

Significance: *p < .05 **p < .001

Sociodemographic characteristics encompassed gender, age, academic qualification, occupation, and domestic environment.

The internal consistency was calculated for all scales used. This can be described as good especially for the item sets at the center of the study:

- *I think it is important that the GP...* (question 14, see Table 1); Cronbachs α = .891
- *The GP I consult in care matters...* (question 15, see Table 1); Cronbachs α = .832

### Recruitment and participants

The survey target group included all kinds of family caregivers. In order to obtain the broadest possible picture of the reality of care in Germany, the inclusion criterion was deliberately kept general. The initial question determines whether at least one relative, friend or neighbour has been regularly supported, cared for or (otherwise) looked after in the last 12 months. In this way, a wide range of different care constellations should be included.

The survey was conducted online using the LimeSurvey tool between January and July 2020 after a pretest. The anonymous survey was posted in a total of seventeen German-language web forums involving or solely focused on family care. This survey design ensured enough outreach to address caregivers often operating in an informal setting. An anonymous online survey raised the chances of truthful responses without pressurising the survey target group to participate [38].

Participants were recruited in health and care-related discussion forums online. The list of forums was selected mostly using search engines such as Google and Bing, but also by researching web directories provided by several specialist organisations. We used certain keywords such as *caregivers*, *family care* and *carers* to search for as many relevant forums as possible. The selected forums were usually embedded in general information portals on the subject of care. These websites are intended to support family caregivers across the board on a wide variety of questions relating to care in a domestic setting (no specific clinical pictures) and enable an exchange.

After that, we contacted the forum admins and asked for permission to recruit for the survey. 17 of the 29 forums gave their permission. A link to the online survey to invite participants was posted in a forum thread stating the study purpose and procedure.

### Data analysis

We analysed the data using SPSS 23.0 for Windows. In addition to the descriptive analysis, a *t*-test with independent samples was used to identify significant differences between two groups. Two levels of a significance were tested for (mean difference at *p* < .05 and *p* < .001). This parametric method has a high test strength and is considered to be statistically robust. The necessary conditions were met with the number of cases, the normal distribution of the groups to be distinguished and the fact that the samples come from the same population [40]. First, the answers to the two item sets used in the questionnaire were compared with a *t*-test (desired support from the GPs vs. experienced support, see Table 1). Subsequently, a cross-tabular breakdown was used to search for items which show highly significant differences (*p* < .001) regarding the general satisfaction with the GP’s support (see Tables 2 and 3).

**Table 2:** Contingency table broken down into satisfaction items with support from general practitioners; columns only contain *completely agree* or *somewhere agree* answer categories

		*The general practitioner I consult in care matters...* (N = 438)		
		*… sees not only the needs of the person in my care but also my opinions, needs and stresses.*	*… tells me about local support and assistance services.*	*… is early to acknowledge me as the caregiver with the awareness that I am responsible for my family member’s care from the beginning.*
How would you rate the support that your GP has given you in caring for your family member?	Very good/ Mostly good	69%**	76%**	59%**
	Mostly not so good/ Not good at all	44%**	21%**	11%**

Significance: **p < .001

**Table 3:** Contingency table; column only shows *completely agree* and *somewhat agree* response categories

		*Someone recently said: “My GP can usually help me out when I ask about care for my family member.” How far does this apply to your GP?* (*N* = 438)
How would you rate the support that your GP has given you in caring for your family member?	Very good/ Mostly good	95%**
	Mostly not so good/ Not good at all	16%**

Significance: **p < .001

We used binary logistic regression analysis at *p* < 0.05 to test for potentially influential factors. Like classic linear regression, binary logistic regression is a method for the statistical explanation of the occurrence of values of the dependent variables that are caused by the influences of one or more independent variables. The peculiarity of binary logistic regression is that it is used for the special situation in which the dependent variable is binary-coded and therefore only has two characteristics. The aim of the study was to identify indications of how different aspects of the experienced support from GPs affect the general satisfaction with the GP’s support. Accordingly, we analysed what influence the individual items regarding personal experience (question 15) could have on satisfaction with GP’s support (question 20). The ordinal independent variables were treated as continuous variables.

All sociodemographic characteristics (gender, age, academic qualification, occupation, and domestic environment) were also included in the analysis.

## Results

### Sample

A total of 612 people who had been caring for at least one family member, friend or neighbour on a regular basis in the last 12 months according to their own statements completed the online survey; another 23 participants took part but did not complete the survey and were not included in the analysis. The sample population was as follows:

- Gender: 93% female, 7% male
- Mean age: 54 (median: 55)
- Academic qualification: 9% lower secondary, 25% upper secondary, 42% high school matriculation grade, 7% university graduation, 17% other academic qualification.
- Occupation: 9% in full-time employment, 51% in part-time employment, 15% retired, 17% not in employment, 8% other

### Living conditions of family caregivers

Of the respondents, 39% provided care alone whereas 61% shared their care responsibilities with another person. The respective care recipient had been given a care level (German: ‘Pflegestufe’^2^) according to 49% and no care level according to 39% of the respondents; 12% did not know. With regard to the duration of care, 22% stated that they had been providing care for less than a year, 34% for one to 2 years, 27% three to 5 years and 17% for 5 years or longer. Care recipients were usually a parent or parent-in-law (54%); followed by husband, wife or life partner (22%); own child, foster child, godchild or child-in-law (14%); or another relative (10%). Of the respondents, 57% were living in the same household as the care recipient.

With respect to the physical condition of the care recipient, 88% indicated severe disabilities whereas 64% responded with severe cognitive disabilities. Care activities focused on assistance and mental stimulation during everyday life at 94%, household chores and assistance in household chores at 84%, assistance in personal care, nutrition and mobility at 79%, and arranging for assistance and care such as filling out and submitting applications and holding appointments with the authorities or doctors at 72%. Medical and nursing activities were carried out by 40% of the respondents.

Of the respondents, 69% stated that the burden on their health in providing care was very severe or moderately severe as opposed to 31% less severe or negligible. Of the former, 84% were providing care activities on their own compared to 59% of those sharing the care activities (*p* < .001). Regarding emotional burden, 58% assessed their own stress from providing care as very severe or moderately severe.

### Importance of the general practitioners and support needs

GPs play a very or moderately important role as contact persons for matters of care according to 67% of the respondents as opposed to 33% for whom GPs played little or no role. Correspondingly, 72% stated that they consulted their own or someone else’s GP in care matters; 39% stated that they would often consult their GP, 15% occasionally, and 18% somewhat seldom.

In line with the research interest, the respondents first received an item set in which they could state which aspects of support from the GP were most important to them. Further on, these items were transferred to the personal experience to reveal the extent to which the respondents’ expectations and desires were reflected in the reality of the support they received. Here, only those caregivers who had consulted their doctor with regard to care were surveyed (*N* = 438).

As shown in Table 1, support from GPs for family caregivers was received favourably in terms of psychosocial support. These results reflect the GP’s approach as contact person familiar with the caregiver’s situation while dedicating a great deal of attention to the person in need of care.

However, there were also weaknesses. For example, the desires of family caregivers for a proactive role from GPs in recognising and anticipating care issues were not always fulfilled. Family caregivers also expressed a desire for a strong advisory role of the GP in arranging the conditions for care as well as legal aspects and information on assistance and support services.

Referring to those respondents that consulted a GP in care matters, 75% saw this as moderately or very important with respect to providing a source for advice and information in organising care. GPs told 61% of these respondents about supporting services at least once. Respondents were mostly told about care services and welfare centres (56%), day centres or short-term care (34%) and assistance for everyday situations (31%).

### Satisfaction with support from general practitioners

In total, 68% of the respondents who consulted a GP in care matters felt very well or moderately well supported compared to 32% who did not feel well supported or supported at all. 70% of the respondents state that the GP can usually be of great help when it comes to a question about care.

Following the comparison of desired support from the GPs and experienced support (see Table 1), a cross-tabular breakdown was used to search for items which show highly significant differences (*p* < .001) regarding the general satisfaction with the GP’s support (see Tables 2 and 3).

Family caregivers who were moderately or very unsatisfied with the support they were given by GPs complained of the lack of three striking support aspects compared to those that were satisfied (see Table 2). These comprised directly addressing and properly considering family caregivers on the one hand, and information on assistance and support services on the other.

A further breakdown confirms this finding. Respondents found the willingness and ability of GPs to provide assistance in organising care crucial to general satisfaction with the support they received from GPs (see Table 3).

### Factors affecting satisfaction with support from general practitioners

The results from binary logistic regression analysis reveal a number of stronger and weaker factors for subjective satisfaction amongst family caregivers with their GPs (see Table 4). For example, the respondents appreciated it when GPs knew about their situation in general and at personal level (Items 1, 2) as well as signalled responsibility with regard to the role they had taken (Item 3). Consideration of individual needs and desires of care recipients also represented an important predictor (Item 8), together with ability to support the caregiver with explanations and ideally mediation between the caregiver and care recipient (Item 20). Information on local advice and assistance services played a major role in the satisfaction felt by the respondents (Item 13). GPs identifying and addressing the caregiver as such early on also counted as an influential factor (Item 21).

**Table 4:** Binary logistic regression, influential factors identified^a^ (*N* = 438)

Dependent variable: Satisfaction with support from general practitioners *(“How would you rate the support that your GP has given you in caring for your family member?”)*										
Independent variable (possible influential factor or predictor) *The general practitioner I consult in care matters...*	Omnibus test (Step 1: Model)	Log-Likelihood	Cox & Snell R^2^	Nagelkerke R^2^	Hosmer-Lemeshow Chi^2^	Coefficient of regression β	Exp (B)	Significance	95% confidence interval Exp (B)	Standard error
1) … is familiar with everyday life and challenges of caring for family members.	.000	403.368	.286	.400	.000	2.889	17.972	.000	10.709; 30.162	.264
2) … is familiar with my personal situation as a caregiver.	.000	431.158	.239	.335	.000	2.522	12.448	.000	7.654; 20.246	.248
3) … feels responsibility for the issues facing people caring for family members and provides advice and assistance.	.000	413.533	.269	.376	.000	3.455	31.659	.000	15.090; 66.422	.378
4) … does not wait for me to raise issues but proactively addresses them.	.037	546.806	.010	.014	.000	.585	1.795	.044	1.017; 3.169	.290
5) … provides emotional support such as by giving encouragement during care.	.000	510.691	.088	.123	.000	1.989	7.309	.000	3.436; 15.548	.385
6) … makes decisions with me on caring for the person in need of it.	.004	542.802	.019	.026	.000	.598	1.818	.004	1.208; 2.736	.209
7) … sees not only the needs of the person in my care, but also my opinions, needs and stresses.	.000	531.878	.043	.060	.000	.916	2.498	.000	1.656; 3.769	.210
8) … considers the person in my care in decisions and responds to his or her requests.	.000	433.072	.236	.330	.000	3.072	21.579	.000	11.093; 41.978	.340
9) ... encourages me to raise my own health concerns.	.000	486.108	.138	.193	.000	3.085	21.861	.000	6.790; 70.391	.597
10) … has enough time for me.	.058	547.502	.008	.012	.000	.442	1.555	.059	.983; 2.462	.234
11) … conducts home visits to relieve me and to make an impression of the person in my care in our home environment.	.000	486.108	.138	.193	.000	3.085	21.861	.000	6.790; 70.391	.597
12) … gives me time to consider important decisions on care.	.122	548.787	.005	.008	.000	.392	1.480	.119	.904; 2.422	.251
13) … tells me about local support and assistance services.	.000	441.421	.221	.309	.000	2.311	10.087	.000	6.299; 16.153	.240
14) … makes arrangements for support and assistance services for me.	.016	545.380	.013	.018	.000	1.299	3.667	.037	1.079; 12.463	.624
15) … advises me on legal aspects such as power of attorney, care assistance, and driving.	.000	522.476	.063	.088	.000	2.343	10.412	.000	3.197; 33.906	.602
16) … conducts regular follow-up observations.	.028	546.346	.011	.015	.000	.464	1.590	.030	1.047; 2.416	.213
17) … makes diagnoses early.	.079	548.086	.007	.010	.000	.360	1.433	.080	.958; 2.143	.205
18) … supports and provides therapy for my family member.	.000	527.205	.053	.074	.000	1.040	2.828	.000	1.842; 4.342	.219
19) … arranges doctors specialised in the field for us.	.000	517.243	.074	.104	.000	2.845	17.208	.000	5.039; 58.767	.627
20) … explains the situation to the person in my care in ways that he or she can understand, therefore supporting me.	.000	467.789	.173	.242	.000	2.694	14.793	.000	7.570; 28.908	.342
21) … is early to acknowledge me as the caregiver with the awareness that I am responsible for my family member’s care from the beginning.	.000	452.523	.201	.281	.000	2.422	11.270	.000	6.378; 19.916	.290

Coding of dependent variables: 0 = Completely agree/Somewhat agree; 1 = Somewhat disagree/Completely disagree

^a^Classification according to Cohen [41]: low or weak explained variation | Nagelkerke R^2 ^| = <.1; medium or moderate explained variation | Nagelkerke R^2 ^| = .13–.3; high or strong explained variation | Nagelkerke R^2 ^| = >.35

No significant differences were found with regard to the sociodemographic variables surveyed. Accordingly, no sociodemographic variables could be identified as influential factors.

## Discussion

### Principal findings and comparison with prior work

The results from the survey showed first of all that GPs act as central contact persons in whom family caregivers place high levels of ability and trustworthiness. A good three out of every four respondents (72%) consulted a GP in care matters, with 54% stating that they did so frequently. This confirms previous studies that emphasised the role of support from GPs in this target group [4, 9, 28]. From the point of view of family caregivers the importance of the GP has further increased in recent years [28].

The results show that it is particularly important to the respondents that GPs feel responsible for the situation of caring relatives, that they can empathise with them and that they are able to see the individual care situation. Accordingly, early identification of caregivers by the doctor is desired. Joint decision-making and targeted referral to support services and specialists are also important to the respondents. From the point of view of a majority of those surveyed, GPs are key players in providing information on care. As a result, by coming to the assistance of family caregivers, GPs play a major role in successful outcomes of family care and long-term stabilisation of family caregivers [5, 26]. In an expert report, the National Association of Statutory Health Insurance Physicians (Kassenärztliche Bundesvereinigung) points out that caring relatives need ‘natural’ contact in the health care system who, in addition to caring for those in need of care, also keeps an eye on the physical health and emotional concerns of those giving help [28, 42]. Due to their position in the German health system, GPs are particularly well suited for this. Other studies show that family caregivers attach great importance to family doctors because they want a permanent, trustworthy contact who can take on advisory and coordinating tasks [24]. From the point of view of the family caregivers, familiarity of GPs with personal care situations, approachability in a variety of issues and directly addressing care recipients proved especially important.

However, the results show that GPs do not always completely match the requirements. For example, GPs did not always show the same level of consideration for the needs – health and otherwise – of the family caregivers as they did for their patients as care recipients. Research literature has addressed a tendency to focus on care recipients while reducing family caregivers to their functional role and marginalising the psychosocial effects of providing care [27, 35–37]. Another problem is that general practice staff do not always identify family caregivers early on for them to be able to involve family caregivers in this role. In this context, one of the difficulties is that general practice staff members are often not sufficiently trained in specific tasks such as identifying and supporting family caregivers [43]. Apart from that, the results of the present study state that only some GPs support family caregivers with information on advice and assistance services. This generally corresponds with the finding that GPs often show insufficient general awareness of external forms of assistance for family caregivers [24, 31] and are not involved in local health networks [44].

The regression analysis has shown that the knowledge of the personal care situation and the ability to mediate between the needs of caregiver and care recipient is important as an influencing factor for subjective satisfaction among family caregivers with their GPs. Especially the advisory activities with regard to Information on local advice and assistance services are of great relevance.

Particularly in this context, there is a need for optimization from the perspective of caregivers. Improving and integrating GPs into other bodies and organisations giving advice and support such as care centres, outpatient mental health services and dementia networks would provide essential leverage to the effectiveness of support given by GPs to family caregivers, who have received little attention from studies up to now. Advice services are especially scarce in rural areas, reducing the number of opportunities that GPs have to refer people affected by home care. However, this kind of cooperation would seem beneficial and urgent. Family members need arrangements for practical everyday assistance, financing opportunities, legal advice, coping strategies and relief in cases of overburden and health deterioration. Arranging assistance networks for patients and family members may substantially reduce the danger of burnout amongst caregivers [31]. However, this would depend on reinforcing interdisciplinary communications between health sectors as well as setting up formal and informal cooperation networks [45, 46] while giving GPs a solid knowledge basis on local advice sources to ensure fast, unbureaucratic referrals.

The results of the present study show that family caregivers are particularly satisfied when GPs consistently refer to offers of help and advice. This corresponds to widespread demands for putting GPs into the role of health mediators, who specifically promote health, refer patients to suitable offers of help and, thus, contribute more to prevention [47]. This also applies to the support and (preventive) stabilisation of family caregivers. Meanwhile, care concepts have been developed to structurally strengthen the GP setting as a contact for this target group. For example, a KBV concept aims to prevent or minimise the health risks and limitations for caregivers [48]. The concept starts with the central key function of general practice and is intended to enable early individual support through a targeted situation analysis and advice. The care concept intends to determine the individual care-related health risk of the caregiver and, on this basis, to offer further advice on physical and psychosocial issues. However, this requires local (health) networks as well as support and (psychosocial) counselling actors with whom GPs actively cooperate [24]. A systematic review by Plöthner et al. points out the importance of strengthening outpatient care structures [49]. The researchers draw the conclusion that establishing an outpatient care system, which supports families and friends in providing (elderly) care, while meeting the needs and wishes of informal caregivers, is of high relevance.

Apart from that, there is some discussion on the introduction of case and support managers to assist GPs in supporting family care situations [50]. One of the benefits may be an improvement in analysis and control of the treatment situation for the care recipient as well as early stabilisation and advice for family caregivers.

### Optimisation approaches

Early involvement of family caregivers is essential towards good care and promotes satisfaction and mutual trust and confidence [51]. Therefore, further training would help GPs to be more aware that addressing the needs of family caregivers is crucial to the long-term success of care in a domestic setting [36, 37, 52, 53]. Practice staff could undergo specific training programmes as well in order to help identify and support (informal) caregivers. Home visits may help understand care issues more effectively.

Stabilisation strategies in consultations with patients and family members should not be underestimated as important skills [31]. GPs could contribute to orientation and calming and otherwise boost resilience in consultations with patients and family members.

Thorough and early information on local assistance and support services would allow family caregivers access to information on organising care in good time [10, 53]. Giving family members the corresponding support may improve outpatient care so as to allow people requiring care to stay within their home setting longer [24, 54].

### Limitations and directions for future research

Due to its general focus, the study should include family caregivers from as many areas as possible and, thus, allow the broadest possible picture regarding the question of GP support. In the course of the study it was possible to recruit a sociodemographically heterogeneous sample of caregivers, in which, among other things, different care constellations, age groups and educational backgrounds are represented (see description of the sample). However, the study is unable to produce a representative body of opinion due to the limited number of cases and the self selection of respondents participating in the survey. In addition, the fact that an online survey was carried out also influences the question of representativeness. In the case of the present work, the recruitment of family caregivers via GPs would have meant a considerably greater expenditure of time and resources. So an online survey of family caregivers was an easy-to-implement solution in which a high number of cases and a heterogeneous respondent group could be recruited.

A comparison with other studies and official statistics shows that the sample does not reflect the actual distribution of sociodemographic characteristics among caring relatives [30]. For example, the spouses or life partners are less represented in the sample, while the group of other relatives is proportionally more represented. It can also be assumed that certain sub-groups within the group of family caregivers are more difficult to reach via an online survey. In this context, it should be noted that older people (70 years and older) are relatively unwilling to take part in online activity, resulting in a relatively low proportion of older family caregivers in the sample population [38]. Since the survey was exclusively placed in German-language web forums, there was a language barrier for people with a migration background. So it is likely that they were not recruited in adequate numbers. Studies have shown that caregivers with a migration background generally have only limited access to sources of advice and information as well as medical support, so that this group is presumably underrepresented in the present study [55].

For the mentioned subgroups of family caregivers it can be assumed that the online survey systematically leads to underrepresentation. Accordingly, it may be assumed that recruiting family caregivers from other settings – such as waiting rooms in general practices – may lead to more generalisable results on family caregivers as a whole. Such studies are warranted especially with regard to optimising general medical practice with regard to the needs of family caregivers.

## Conclusions

Family caregivers play a vital role, as caring for infirm people reliant on care in a domestic setting would not be possible without them. GPs are essential in supporting them, especially when providing information on planning and organising care as well as advice in other fields alongside psychosocial support.

The results show that family caregivers place GPs in a special role of supporting. From respondents’ past experience, GPs live up to their responsibility in different ways. Even so, there are also indications that general practices have not always been successful in satisfying all the needs of family caregivers.

We recommend that GPs undergo further training to reinforce awareness that the care triad of needs, requirements and stresses amongst family caregivers also plays a vital role in care outcomes. With this in mind, general practice staff should adopt a pre-emptive strategy towards approaching family members about potential issues and informing them about existing assistance and support services.

## Supplementary Information

**Additional file 1.** Questionnaire (translation).

## Data Availability

All data generated or analysed during this study are included in this published article.

## Abbreviation

GP(s): General Practitioner(s)

## Footnotes

1 The focus is on targeted support from GPs with outpatient case and crisis management guided by care experts and adapted to need. Apart from that, *DemStepCare* aims to raise awareness and capability in dealing with dementia amongst GPs in a comprehensive approach.

2 The extent of services which the person requiring care is entitled to depends on the class of nursing care.
