# Supplementary material for: Support, needs and expectations of family caregivers regarding general practitioners – results from an online survey
Source: BMC Fam Pract. 2021 Mar 3;22:47. doi: 10.1186/s12875-021-01381-4 (PMC7927394; doi:10.1186/s12875-021-01381-4)
Supplement: Supplementary file 1 — Additional file 1. Questionnaire (translation). [file 12875_2021_1381_MOESM1_ESM.docx]

**1. Have you regularly cared for, nursed or otherwise supported a friend, family member or neighbour in the last twelve months?**

⃝ Yes ⃝ No **=>** End survey

**2. Have you provided this support or care on your own or shared the responsibility with others?** (not including care services and welfare centres such as those provided by care insurance)

⃝ On my own ⃝ Shared with others

**3. Has the person you have been caring for been given a care level?**

⃝ Yes ⃝ No ⃝ Don’t know

**4. How long have you been helping this person?**

⃝ Less than a year ⃝ One to three years ⃝ 3-5 years ⃝ 6-8 years ⃝ More than 8 years

**5. What is the relationship between you and the person you have been caring for?**

⃝ Husband, wife or life partner

⃝ Parent or parent in law

⃝ Own child, foster child, godchild, child in law

⃝ Other relative such as uncle, aunt, brother, sister or grandchild

⃝ Boyfriend or girlfriend

⃝ Neighbour

⃝ Other

**6. In your opinion: How far does physical disability affect the person you are caring for?**

⃝ Very severely ⃝ Moderately ⃝ Slightly ⃝ Not at all

**7. How far does mental disability affect the person you are caring for?**

⃝ Very severely ⃝ Moderately ⃝ Slightly ⃝ Not at all

**8. Are you currently living in the same household as the person you are caring for?**

⃝ Yes ⃝ No

**9. Which of the following points apply to the type of care you are providing?**  (Cross as many as apply)

⃝ Personal care, diet and mobility such as helping with showering, eating, changing beds, moving or toileting

⃝ Medical or nursing care such as measuring blood pressure, dressing wounds, applying compression stockings or giving medication

⃝ Household chores such as cleaning, cooking, washing and shopping

⃝ Arranging for care and assistance such as submitting applications, coordinating care services, or help in visits to doctors or public authorities

⃝ Everyday care and mental stimulation for the person you are caring at home and away, such as providing company or arranging joint activities

⃝ Other

**10. How much stress is it for you to you to provide care to the person in need of it?**

Physical and health effects: ⃝ Severe ⃝ Moderate ⃝ Slight ⃝ Negligible

Emotional and mental effects: ⃝ Severe ⃝ Moderate ⃝ Slight ⃝ Negligible

**11. Generally speaking: How important is the GP as a contact person for questions about caring for your family member?**

⃝ Very important ⃝ Moderately important ⃝ Not very important ⃝ Not important at all

**12. Do you consult your GP or another GP on the care you are giving to your family member?** By this we do not necessarily mean your own GP, but rather the GP that you consult on caring for your family member. This could also be your family member’s GP.)

⃝ Yes ⃝ No, never **=>** Please go straight to **question 14**

**13. How often do you consult your GP or another GP about the care you are giving to your family member?**

⃝ Often ⃝ Sometimes ⃝ Rarely

**14. People caring for family members can have very different needs and expectations as to how a GP should support them. How important are the following points to you personally?**

| *I think it is important that the GP...* | Very important | Moderately important | Not very important | Not important at all |
| --- | --- | --- | --- | --- |
| … is familiar with the everyday life and challenges of people caring for family members, is familiar with the topic at hand, and is capable in this field. | ⃝ | ⃝ | ⃝ | ⃝ |
| … is familiar with my personal situation as a caregiver. | ⃝ | ⃝ | ⃝ | ⃝ |
| … feels responsibility for the issues facing people caring for family members and provides advice and assistance when I have difficulties. | ⃝ | ⃝ | ⃝ | ⃝ |
| … does not wait for me to raise issues with the GP but proactively addresses issues. | ⃝ | ⃝ | ⃝ | ⃝ |
| … provides emotional support such as encouragement during care. | ⃝ | ⃝ | ⃝ | ⃝ |
| … makes decisions with me on caring for the person in my care. | ⃝ | ⃝ | ⃝ | ⃝ |
| … sees not only the needs of the person in my care but also my opinions, needs and stresses. | ⃝ | ⃝ | ⃝ | ⃝ |
| … includes the person in my care in decisions and responds to his or her requests. | ⃝ | ⃝ | ⃝ | ⃝ |
| … encourages me to raise my own health concerns. | ⃝ | ⃝ | ⃝ | ⃝ |
| … has enough time for me. | ⃝ | ⃝ | ⃝ | ⃝ |
| … performs home visits to relieve me and get to know the person in my care in our home environment. | ⃝ | ⃝ | ⃝ | ⃝ |
| … gives me time to consider important decisions on care. | ⃝ | ⃝ | ⃝ | ⃝ |

| *I think it is important that the GP...* | Very important | Moderately important | Not very important | Not important at all |
| --- | --- | --- | --- | --- |
| … tells me about local support and assistance services that I might be able to use for support, such as short-term care services, care centres and self-help groups. | ⃝ | ⃝ | ⃝ | ⃝ |
| … makes arrangements for support and assistance services for me. | ⃝ | ⃝ | ⃝ | ⃝ |
| … advises me on legal aspects such as power of attorney, care assistance, and driving. | ⃝ | ⃝ | ⃝ | ⃝ |
| … conducts regular follow-up observations on how far the mental and physical situation of my family member has progressed. | ⃝ | ⃝ | ⃝ | ⃝ |
| … makes diagnoses early enough for us to have clarity and prepare for further developments such as deterioration in health condition. | ⃝ | ⃝ | ⃝ | ⃝ |
| … manages and provides medical treatment to my family member. | ⃝ | ⃝ | ⃝ | ⃝ |
| … arranges doctors specialised in the field for us. | ⃝ | ⃝ | ⃝ | ⃝ |
| … explains the situation to the person in my care in ways that he or she can understand, therefore supporting me in my care efforts. | ⃝ | ⃝ | ⃝ | ⃝ |
| … is early to acknowledge me as the caregiver with the awareness that I am responsible my family member’s care. | ⃝ | ⃝ | ⃝ | ⃝ |

**Please only answer the following questions if you have already spoken to your GP about care. Otherwise go straight to page 8, question 24.**

**15. Which of the following statements is correct in your experience?**

| *The GP I consult in care matters...* | Completely agree | Somewhat agree | Somewhat disagree | Completely disagree |
| --- | --- | --- | --- | --- |
| … is familiar with the everyday life and challenges of people caring for family members, and is familiar with the topic at hand and is capable in this field. | ⃝ | ⃝ | ⃝ | ⃝ |
| … is familiar with my personal situation as a caregiver. | ⃝ | ⃝ | ⃝ | ⃝ |
| … feels responsibility for the issues facing people caring for family members and provides advice and assistance when there are difficulties. | ⃝ | ⃝ | ⃝ | ⃝ |
| … does not wait for me to raise issues but proactively addresses them. | ⃝ | ⃝ | ⃝ | ⃝ |
| … provides emotional support such as by giving encouragement during care. | ⃝ | ⃝ | ⃝ | ⃝ |
| … makes decisions with me on caring for the person requiring care. | ⃝ | ⃝ | ⃝ | ⃝ |
| … sees not only the needs of the person in my care but also my opinions, needs and stresses. | ⃝ | ⃝ | ⃝ | ⃝ |
| … considers the person in my care in decisions and responds to his or her requests. | ⃝ | ⃝ | ⃝ | ⃝ |
| … encourages me to address my own health concerns. | ⃝ | ⃝ | ⃝ | ⃝ |
| … has enough time for me. | ⃝ | ⃝ | ⃝ | ⃝ |
| … performs home visits to relieve me and get to know the person in my care in our home environment. | ⃝ | ⃝ | ⃝ | ⃝ |
| … gives me time to consider important decisions on care. | ⃝ | ⃝ | ⃝ | ⃝ |
| *The GP I consult in care matters...* | Completely agree | Somewhat agree | Somewhat disagree | Completely disagree |
| … informs me about local support and assistance services that I might be able to use for support such as short-term care services, care centres and self-help groups. | ⃝ | ⃝ | ⃝ | ⃝ |
| … makes arrangements for support and assistance services for me. | ⃝ | ⃝ | ⃝ | ⃝ |
| … advises me on legal aspects such as power of attorney, care assistance, and driving. | ⃝ | ⃝ | ⃝ | ⃝ |
| … conducts regular follow-up observations on how far the mental and physical situation of my family member has progressed. | ⃝ | ⃝ | ⃝ | ⃝ |
| … makes diagnoses early enough for us to have clarity and prepare for further developments such as deterioration in health condition. | ⃝ | ⃝ | ⃝ | ⃝ |
| … manages and provides medical treatment to my family member. | ⃝ | ⃝ | ⃝ | ⃝ |
| … arranges doctors specialised in the field for us. | ⃝ | ⃝ | ⃝ | ⃝ |
| … explains the situation to the person in my care in ways that he or she can understand, therefore supporting me in my care efforts. | ⃝ | ⃝ | ⃝ | ⃝ |
| … is early to acknowledge me as the caregiver with the awareness that I am responsible my family member’s care from the beginning. | ⃝ | ⃝ | ⃝ | ⃝ |

**16. Someone recently said: “My GP can usually help me out when I ask about care for my family member.” How far does this apply to your GP?**

⃝ Completely agree ⃝ Somewhat agree ⃝ Somewhat disagree ⃝ Completely disagree

**17. What role does your GP play when you are looking for information on care, such as which services are available or who you can ask for further advice?**

⃝ Very important ⃝ Moderately important

⃝ Not very important ⃝ Not important at all

**18. How often has your GP told you about supporting services?** (By this, we don’t necessarily mean actual local services but more generally that these services are available.)

⃝ Several times ⃝ Once ⃝ My GP has not told me about them.

**19. Which of the following services has your GP told you about?** (Cross as many as apply)

⃝ Services for assistance in everyday life and everyday living assistance ⃝ Day centres or short-term care

⃝ Self-help groups ⃝ Care services and welfare centres ⃝ Advice on care and care centres

⃝ Dementia networks as well as contact points specialised in dementia

⃝ Care, nursing and retirement homes ⃝ Other

**20. How would you rate the support that your GP has given you in caring for your family member?**

⃝ Very good ⃝ Mostly good ⃝ Mostly not so good ⃝ Not good at all

**21. What satisfied you the most?** A few key words would be enough.

**__________________________________________________________________________________**

**__________________________________________________________________________________**

**__________________________________________________________________________________**

**22. And what left you the most dissatisfied?** A few key words would be enough.

**__________________________________________________________________________________**

**__________________________________________________________________________________**

**__________________________________________________________________________________**

**23. What would you as a caregiver expect more in the way of support from your GP?** A few key words would be enough.

**__________________________________________________________________________________**

**__________________________________________________________________________________**

**__________________________________________________________________________________**

**24. Have you ever changed your GP out of dissatisfaction with his or her support in caring for your family member?**

⃝ Yes, this is has happened ⃝ No, this hasn’t happened

*Finally, we would like to ask you for some information for statistical purposes. As with the rest of the questionnaire, the information you give will of course be treated in strict confidence and anonymity.*

**You are…**

⃝ Male ⃝ Female ⃝ Diverse

Your **age**: _____

**What is your highest level of education?**

⃝ “Volks-/Hauptschule” (lower secondary) ⃝ “Realschule” (upper secondary)

⃝ “Abitur”/”Fachabitur” (matriculation grade) ⃝ College or university degree

⃝ Other academic qualification ⃝ No academic qualification

**Are you in employment?**

⃝ Yes, full time ⃝ Yes, part time

⃝ Retired ⃝ Not in employment

⃝ Job seeker ⃝ Other

**What best describes your place of residence?**

⃝ Large town or city (pop. > 100,000) ⃝ Medium-sized town (pop. 20,000-100,000)

⃝ Small town (pop. 5,000 to 20,000) ⃝ Village (less than 5,000)
